# Effect of anchor shear deformation on the propagation rules of excitation stress waves

**DOI:** 10.1038/s41598-024-81755-7

**Published:** 2024-12-02

**Authors:** Chuanming Li, Chuan Li, Bochao Nie, Qinghua Han, Jianguo Cao

**Affiliations:** School of Mining Engineering, Anhui University of Science & Technology, Huainan City, 232000 Anhui Province China

**Keywords:** Stress-wave nondestructive testing, Anchor shear deformation, Anchor solid damage, Energy loss ratio, Civil engineering, Mechanical engineering, Metals and alloys

## Abstract

To address the shear deformation failure resulting from misalignment of rock strata in anchor support, a range of methods, including laboratory tests, numerical simulation, and others, are employed to study the deformation process of anchor rods under shear comprehensively. This approach permits an investigation of the deformation characteristics of the surrounding rock and anchoring agent under shear. Moreover, the propagation characteristics of the stress wave in the anchor rods under shear with varying degrees of deformation have been elucidated. A novel non-destructive testing method for assessing the degree of shear deformation in anchor rods is proposed based on the correlation between the energy evolution law of the stress wave and the degree of anchor deformation. Following experimental verification, it was determined that the average discrepancy between the detection outcomes of the stress wave method and the actual measurement results was minimal and consistent. The findings provide a theoretical foundation for the non-destructive testing of anchor rod shear deformation under on-site support conditions.

## Introduction

In the field of infrastructure engineering, anchor anchoring represents a pervasive and fundamental engineering technology^1^. Anchor support can stabilize a discontinuous rock body, rock layer, or gravel layer, forming a high-strength and stable whole. It can also serve as a suspension, combined beam, or reinforcement, thereby providing support^2^. However, a considerable number of weak structural surfaces are present within the rock mass, which are susceptible to deformation, sliding, and shear forces, ultimately leading to structural deterioration. In excavation projects with slopes, the rock body is susceptible to shear slip along structural surfaces, which can result in collapse and landslides^3^. In the context of underground engineering construction, the occurrence of lateral solid deformation between the surrounding rocks can result in significant damage to the underground engineering structure^4^. In the context of deep, buried roadway support in mines, the roadway is susceptible to bottom dropsy, which is accompanied by two gangs of rock shear slip deformation^5^. The anchoring of the rock body by surrounding rock shear is prone to radial shear deformation and shear damage, which can ultimately result in anchorage failure^6^. A substantial body of engineering evidence demonstrates that anchor shear damage represents a significant proportion of anchor anchorage failure.

Therefore, many scholars have studied the shear performance of anchor rods, and Pellet & Egger^7^ have found that the anchor rods will produce plastic hinge at the maximum bending moment as well as damage by establishing the elastic foundation beam model of anchor rods of nodular rock body under the action of shear load. Zhang & Liu^8^ found that the plastic hinge occurs after the anchor yields damage by observing the anchor deformation characteristics, and the deformation and damage of the anchor are related to the anchorage angle and the type of surrounding rock, when the surrounding rock is hard, the smaller the anchorage angle is, the better, and vice versa, the larger, the better. Lin et al.^9^ carried out straight shear experiments by changing the grouting state, the number of anchor rods, and the tilt angle of the anchor rods, and the results showed that grouting in the joints can improve the stress state of the anchor rods, increase the yield displacement of the anchor rods, and co-ordinate the deformation of the grouting body and the anchor rods, to improve the shear strength of the anchor joints. Current research shows that anchor shear deformation is related to factors such as shear location, surrounding rock type, anchorage angle, shear displacement, etc. Therefore, it is possible to detect certain characteristic parameters to determine whether shear damage has occurred in the anchor. However, conventional means of detection, only through the degree of deformation of the surrounding rock^10^, anchor preload changes^11^ in the anchor in what deformation state, the detection process exists hysteresis, unpredictable and cumbersome process, and many other shortcomings, and will cause irreversible damage to the anchor rock body, and even the structure of the safety and stability of the structure has a significant impact on the non-destructive testing (NDT) of anchors began to enter the field of vision of experts and scholars.

Anchor NDT technology is mainly based on stress wave detection and acoustic wave detection, and Mori et al.^12^ have conducted experimental research on artificial defective concrete specimens containing different lengths and depths by adopting non-contact equipment to generate impact and monitor the response and put forward the dynamic response characteristics of the defective concrete structure under the action of impact load, Chaki & Bourse^13^ have monitored the change process of stress level of prestressing strand by selecting ultrasonic guided waves of suitable frequency, and demonstrated the applicability of ultrasonic guided wave procedure to monitor the stress level of strand, Beard & Lowe^14^ have proposed to adopt ultrasonic guided wave guidance to solve the problem of monitoring pullout damage of anchor rods, and verified that the ultrasonic guided wave monitoring technology is able to determine the length of the anchor rods, identify the main defects, through the laboratory experiments and on-site validation experiments,Zhao et al.^15^ study the elastic stress waves propagating along the anchor by generating excitation signals at the exposed end of the anchor, analyze the reflection signals generated by the stress waves between the bottom of the anchor and the surrounding medium, and determine the degree of densification of the anchoring enclosure; Amerini & Meo^16^ develop a reliable index capable of assessing the health of the bolt structure in terms of its loosening/tightening state based on the linear and nonlinear acoustic ultrasound parameters, by which the measurement of the proposed index, the torque applied to the bolt can be known, and thus the health state of the bolt structure can be assessed, Ding et al.^17^ proposed a method to measure the stress of tightened bolts using electromagnetic ultrasonic transducers, and by analyzing the ray paths of the longitudinal and shear waves, the relationship between the axial stresses of the anchors and the time-of-flight ratios of the two modal waves was derived, and based on this, an electromagnetic ultrasonic tester can be used to measure the stresses of the bolts without measuring the axial stress of anchor rods without loosening the bolts. In summary, the current research direction of anchor NDT mainly focuses on three aspects: anchor length testing, anchorage quality testing, and anchor axial force testing, while the research on NDT of structural deformation of anchor under shear is not yet mature.

A substantial body of research has demonstrated that the primary cause of anchor solids shear failure is the shear deformation of anchor rods. This paper will examine the shear damage of anchor solids, proceed to the design of an experimental system for NDT of anchor shear, and conclude with the identification of deformation. Furthermore, the damage characteristics of the anchor rods, anchors, and peripheral rocks under the shear deformation of anchor solids are investigated through the adoption of laboratory tests, numerical simulations, and experimental verification. Additionally, the propagation law of the stress wave under the shear deformation of anchor rods is the subject of particular focus. By employing nonlinear stress wave signal noise reduction preprocessing technology, this study examines the deformation characteristics of anchors subjected to shear forces and the propagation characteristics of stress waves within shear-deformed anchors. A NDT method for evaluating anchor shear deformation is proposed based on the excitation reflection stress wave approach.

## Anchor solid shear damage characterization

### Anchor solid shear experimental system and experimental program

**Fig. 1:**
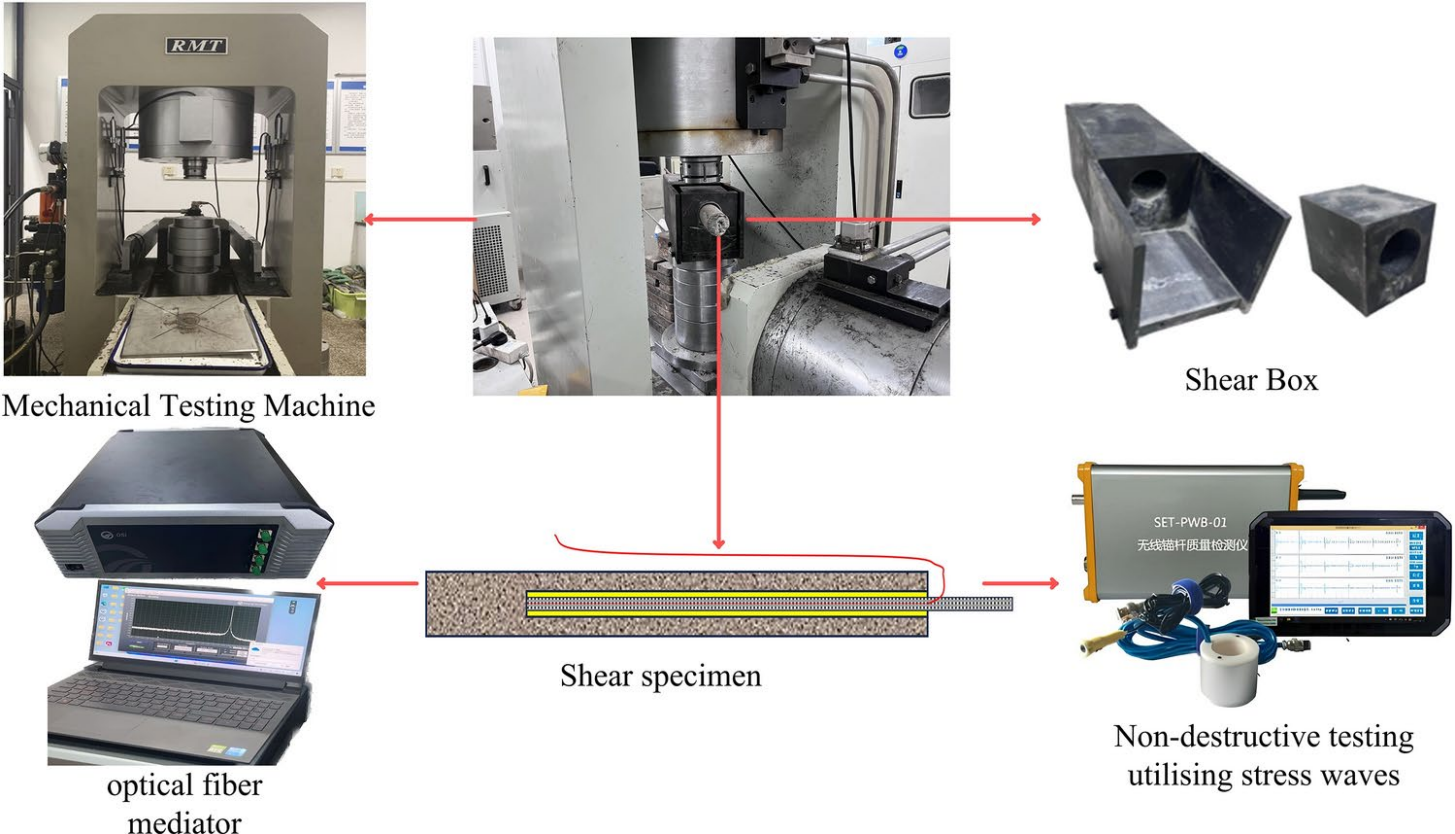
Anchor solid shear non-destructive testing system.

In order to study the deformation characteristics of anchor solid under shear and the stress wave propagation law in the anchor, an experimental system for nondestructive testing of anchor solid in shear was designed as shown in (Fig. 1). The system mainly consists of three parts: anchor shear experimental system, stress wave detection system, and distributed fiber optic sensor detection system. The shear experimental loading equipment by the RMT-150C rock mechanics testing machine and shear experimental loading mold, which shear experimental loading mold contains a rigid fixed frame and shear box. Limited by the size of the shear box, the parameters of the anchor solid specimen for this shear experiment are Φ50 × 500 mm, anchor hole Φ20 × 400 mm, anchoring agent is epoxy resin anchoring agent, and the anchor rod model is HRB335 with the size of Φ10 × 1000 mm. Moreover, referring to the GB50010-2010(2015) concrete structure design specification, C40 concrete was designed to simulate the shear surrounding rock, with the ratio of cement: water: sand: gravel = 10:3.9:12.9:28.8 (KG). The basic working principle of the stress wave detection system is stress wave generated by the vibration of the roll exciter in the rod head; compared with other types of sensors, accelerometers are extremely sensitive to vibration, can pick up the vibration, and will be converted into an electrical signal output time domain waveform. Acceleration sensor model ULT2108, 20 kHz piezoelectric acceleration sensor, sensitivity 1mv, frequency range 0.5 ~ 6000HZ, resonance frequency of 20 kHz. This paper focuses on the study of the stress wave type of elastic wave; due to the uniform texture of the anchor rod and elastic properties of the same, the material vibration in the low-frequency range of stability is good to facilitate the stability of the measurement data. The distributed fiber optic sensor detection system mainly comprises an OSI control software operating system and a high-precision distributed optical frequency domain strain temperature analyzer. High-precision distributed optical frequency domain strain temperature analyzer through the data line connected to the computer and through the channel connected to the distributed fiber-optic sensors, experiments using fiber-optic sensors for the diameter of 0.9 mm high transfer tightly wrapped sheath distributed strain sensing fiber optic cable, which consists of continuously distributed fiber-optic sensing units of equal length, there is no spacing between the adjacent sensing units, real-time collection of high-precision strain data of the anchored solid. The distributed fiber-optic sensor detection system mainly comprises an OSI control software operating system and a high-precision distributed optical frequency domain strain temperature analyzer. High-precision distributed optical frequency domain strain temperature analyzer connected to the computer through the data line and through the channel connected to the distributed fiber optic sensors, experiments using fiber optic sensors for the diameter of 0.9 mm high transfer tightly wrapped sheath distributed strain sensing fiber optic cable, which consists of a continuous distribution of equal length fiber optic sensing units, there is no spacing between the adjacent sensing units, which allows real-time collection of high-precision strain data of the anchor solid. In order to facilitate the collection of data by the distributed fiber optic detector and stress wave detector, the anchor should be pre-treated by pasting the fiber optic and grinding the anchor end section.

Fourteen specimens were set up in this experiment, and the experiment was conducted by applying shear displacement to the anchor solid through the anchor solid shear experiment system. Yin et al.^18^ have proposed that the loading rate will change the damage pattern of rock materials, increasing the loading rate will cause the mechanical properties of the material to show great pseudo-enhancement, and the material will be more broken, so the comprehensive consideration of the shear rate will be set to 0.01 mm/s. Control roller shaker in the distance from the anchor end 10 cm to launch the stress wave through the acceleration sensor in the anchor end cross-section to collect and transmit data while controlling the optical fiber detector to collect position-strain data. In order to ensure the accuracy of the stress wave signal, the experimental sampling rate was set to the maximum sampling rate of 1 MHz, and the number of sampling points was set to 6 K. The experimental system collected and recorded 6 groups of data every 5 mm shear displacement, and the maximum shear displacement was 40 mm.

### Characterization of anchor solid shear deformation

**Fig. 2:**
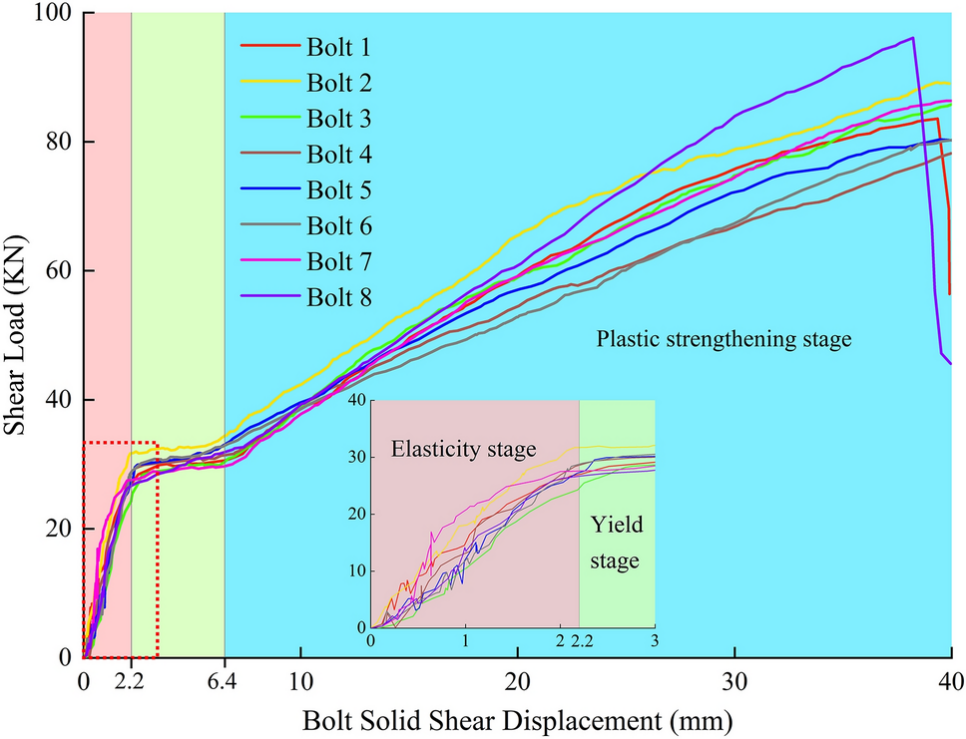
Shear displacement graph.

**Fig. 3:**
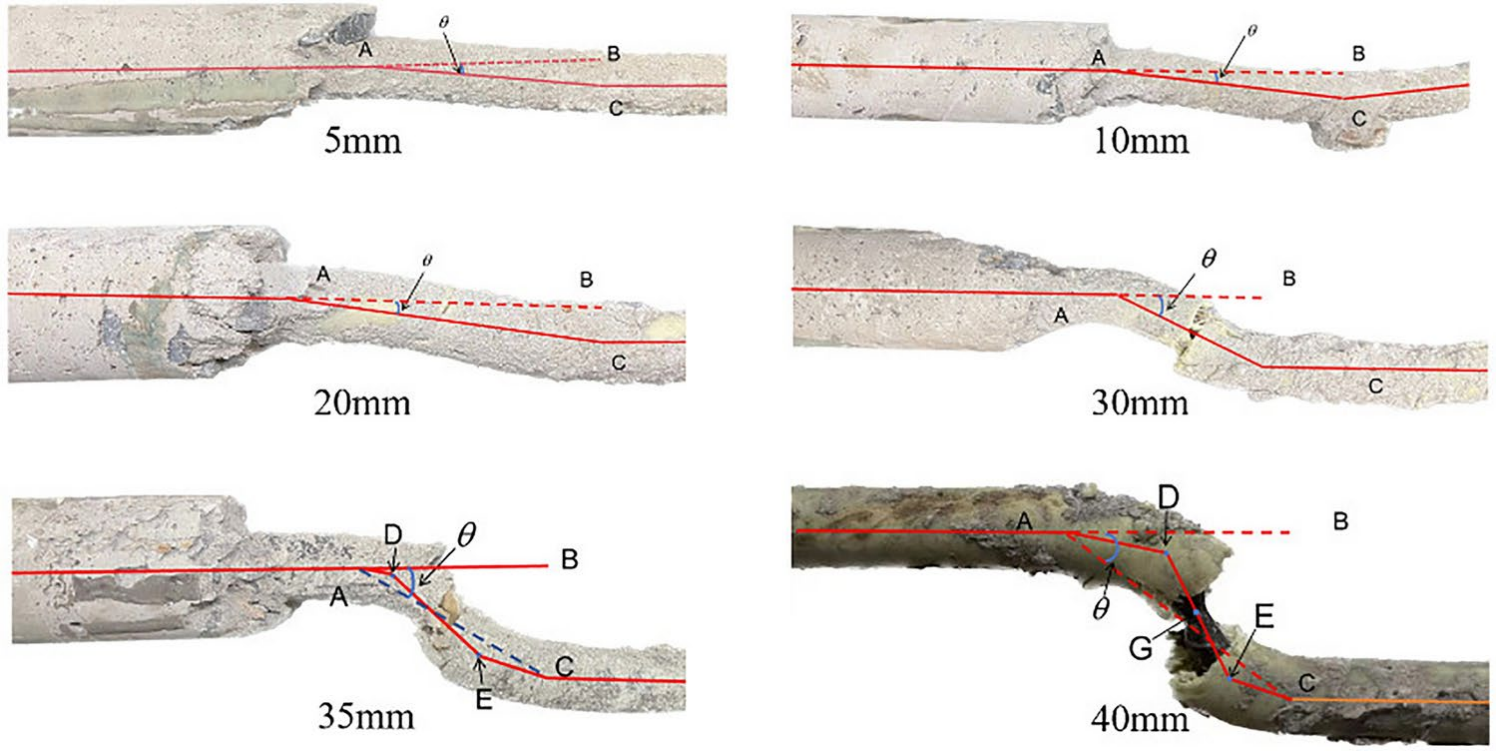
Anchor solid shear deformation diagram.

As shown in Fig. 2 for the eight groups of anchor solid shear experiment displacement-shear force graph, combined with Fig. 3 anchor solid shear deformation graph can be found, Bolt1-8 are there are obvious three stages: elastic stage, yielding stage and plastic strengthening stage. Shear displacement 0–2.2 mm is the elastic stage, the anchor shear force increases rapidly, at this time the anchor mobilises the strength of the surrounding rock to resist the tensile force and shear force; when the shear displacement of 2.2–6.4 mm is the yielding stage, the shear force grows slower, showing a gentle low growth curve, the surrounding rock outside the anchor solid begins to appear a lot of broken and detached from the anchor, at this time the shear force is mainly borne by the low strength broken rock; the shear displacement over After the shear displacement exceeds 6.4 mm, the anchor enters the plastic strengthening stage, the shear force increases linearly, at this time the shear force is borne by the anchor rod, the anchor rod under the action of the shear force undergoes bending deformation, of which the Bolt1 and Bolt8 plastic strengthening stage is shorter, the anchor rod necks after reaching the yield stiffness, and breaks after maintaining a period of shear displacement.

**Fig. 4:**
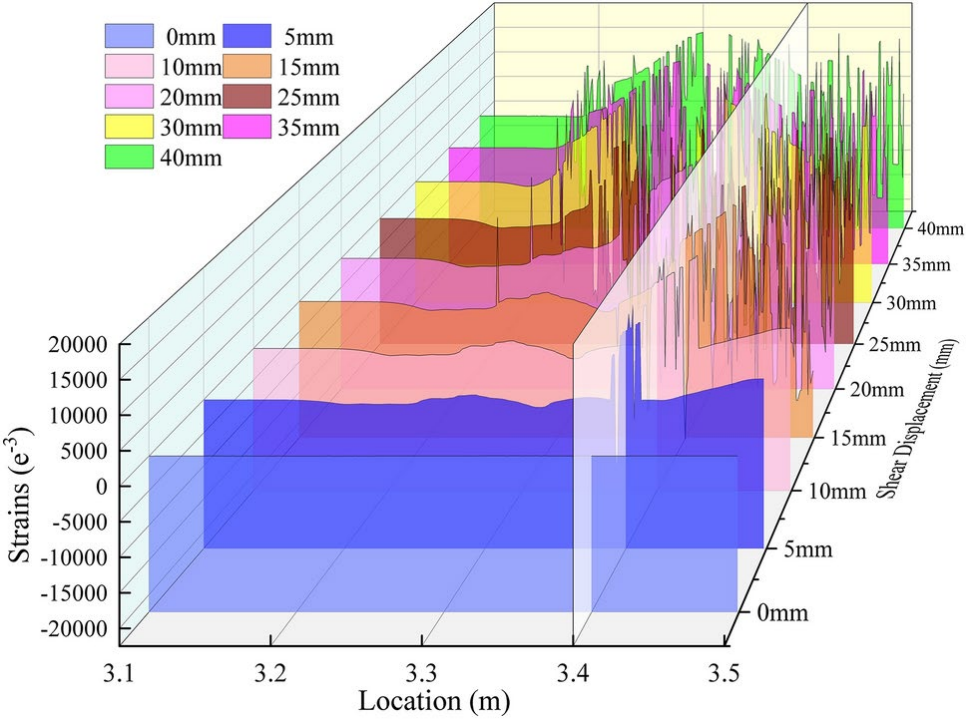
Strain distribution inside the anchor solid under different shear displacements.

As illustrated in Fig. 4, the fiber optic detector is employed to evaluate the internal strain distribution of the anchor solid under varying shear displacements. The section measuring 300 to 400 mm represents the shear position. As illustrated in the figure, when the shear displacement of the anchor solid reaches 5 mm, it becomes evident that the fiber optic sensor malfunctions at the interface between the nodal plane and the extruded deformation section. This indicates that the surrounding rock has been compromised at this juncture. Moreover, the anchors display bending and deformation, and the damage to the fiber optic sensor advances towards the base of the anchors in the nodal plane when the shear displacement reaches 5 to 10 mm. Moreover, the damage to the fiber optic sensor broadens in scope due to the increased displacement and shear force. As a result of the increased displacement and shear force, the area of the nodal plane in close proximity to the interface between the nodal plane and the extruded deformed section begins to expand the damage range through the application of tensile and compressive forces. At a shear displacement of 15 mm, the distributed optical fibers within the nodal plane are destroyed, and the elastic deformation section and the extrusion deformation section also begin to fail. Upon examination of the remaining signals, it becomes evident that as shear displacement increases, deterioration of the fiber optic sensors is observed, progressing from the nodal plane to the elastic deformation section. In conclusion, all fiber optic sensors are destroyed after a displacement of 3.22 m at a truncation displacement of 40 mm.

The preceding analysis demonstrates that the damage to the surrounding rock of the anchor solid, subjected to shear, and the deformation of the anchor rod anchorage commences at the interface between the nodal surface and the extruded deformation section. After that, the damage progresses to the nodal surface, and the extruded deformation section becomes damaged only after the essential destruction of the nodal surface. Ultimately, the damage extends to the elastic deformation section. In the initial stages of shear displacement, the load is primarily supported by the anchorage and surrounding rock. There is a discernible lag in anchor deformation. As shear displacement increases, the shear force is predominantly borne by the anchor until it reaches the plastic stage. The subsequent necking phenomenon ultimately fails the anchor solid as a whole.

### Numerical simulation study on shear deformation characteristics of anchor solid

**Fig. 5:**
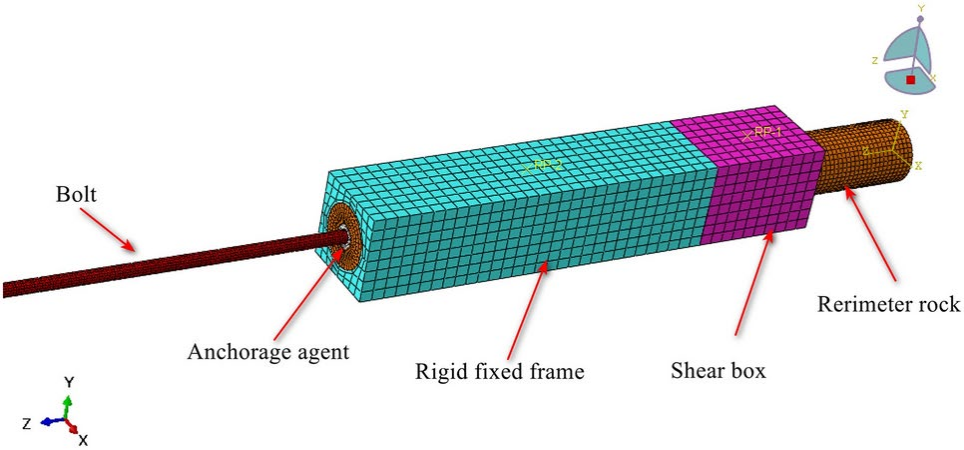
Anchor solid shear model meshing diagram.

In order to verify and supplement the findings of the anchor shear deformation characteristics of the anchor bar and the damage process of the anchor solid derived from the analysis of the results of the anchor solid shear test, a numerical simulation using ABAQUS is employed to observe the damage process of the anchor solid and the changes of the anchor bar in the anchor body under the action of shear. ABAQUS is one of the most advanced finite element analysis programs with powerful analytical capability, which can simulate various physical fields, accurately analyze the structural stress state under complex loads, and solve highly nonlinear problems. In this paper, ABAQUS/Explicit module is mainly used to carry out numerical simulation experiments of anchor shear in order to ensure the unity of experimental and simulation data; the simulation materials in this paper use the same parameters as the experimental materials, the perimeter rock uses the concrete elastic-plastic principal structure, the concrete plastic damage is set to simulate the damage process of the perimeter rock, and the anchor rods and anchors use the conventional elastic-plastic principal structure. As illustrated in Fig. 5, the mesh delineation diagram for the anchor solid shear model, the simulation is configured as a rigid frame with complete fixation. The upper surface of the shear box is subjected to a displacement load in the −y direction, with a load according to the experimental program of 0 ~ 40 mm linear loading.

**Fig. 6:**
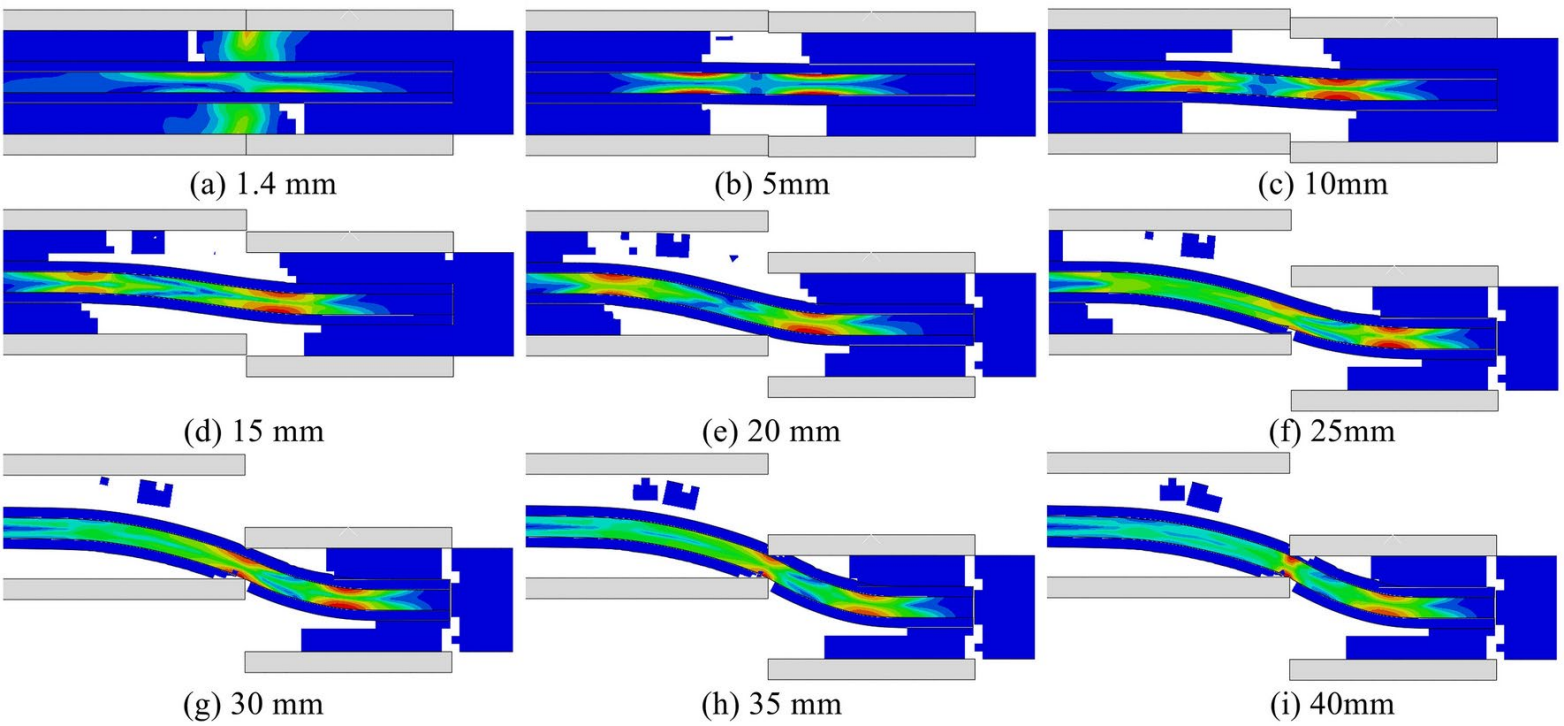
Anchor Solid shear equivalent force clouds.

Shown in Fig. 6 is the equivalent stress cloud diagram of the shear process; analysis of the cloud diagram can be found in the initial loading stage, where the stress is transferred to both sides of the anchor solid by the interface between the nodal surface and the extrusion deformation section, and at this time, the surrounding rock bears most of the stress. Shear 5 mm, due to the shear box and fixed frame contact surface near the surrounding rock are rupture failure, the peak stress appears to decline, the surrounding rock at the stress transfer to the anchor rod, the anchor rod appeared in two sections of the stress concentration point, it can be clearly seen that the anchor rod undergoes a slight deformation; with the increase of the shear displacement, the stress continues to move from the contact surface along the anchor rod to the two sides, the peak stress continues to rise, the anchor solid in the unanchored perimeter of the rock is subjected to stress When the shear displacement of the anchor solid reaches 30 mm, due to the destruction of all the surrounding rocks inside the shear box, a small part of the surrounding rocks bonded by the anchoring agent has not yet been dislodged, and the anchoring agent at the connection between the shear box and the fixed frame appears to be fractured, and the stress is mainly distributed in the joints of the surrounding rocks inside the anchor solid and the connection between the shear box and the fixed frame.

Table 1 compares the shear displacement-bending angle of the anchor solid from laboratory experiments and numerical simulations, which shows that: there is a high degree of uniformity between the numerical simulation and the laboratory, and the relationship of the anchor solid shear displacement with the anchor bar shear displacement and the anchor bar bending The relationship between anchor solid shear displacement and anchor shear displacement and anchor bending angle is not a simple linear increase. Before the anchor solid shear displacement reaches 20 mm, the change of anchor bar shear displacement and anchor solid shear displacement is always different. This is due to the existence of the reduction effect of the surrounding rock; the surrounding rock bears part of the shear force, and the anchor bar shear displacement is smaller than the anchor solid shear displacement. After the anchor solid shear displacement is 20 mm, the relationship curve between anchor solid and anchor shear displacement is linear, and the change is the same, which indicates that the reduction of surrounding rock reaches the limit at this time. At the same time, the change of anchor solid shear displacement-anchor bending angle is more complicated; the curve as a whole shows a slow-fast-slow trend of change; in the anchor solid shear displacement is less than 20 mm, the anchor bending angle change growth is slower, in the anchor solid shear displacement in 20–30 mm, the anchor bending angle is faster, more than 30 mm, the bending angle of the anchor rod and then begin to slow down..

**Table 1:** Numerical simulation test and anchor body shear test bolt deformation table.

Shear displacement/mm	Experimental bending angle/°	Numerical simulation of bending angle/°	Experimental anchor shear displacement/mm	Numerical simulation of anchor shear displacement/mm
0	0	0	0	0
5	3	2.8	4.1	3.7
10	5	5.5	8.7	8.3
20	10	9.2	17.4	17.9
30	24	24.7	27.4	28.2
35	26	25.3	32.5	33
40	30	29.6	37.5	37.2

## Characterization of stress wave propagation in anchors under shear action

The study above demonstrates that the deformation damage characteristics of the surrounding rock, anchor solid, and anchor rods under shear deformation of the anchor solid are distinct. The primary deformation periods for the surrounding rock and anchors are the initial and final shear periods. However, these periods cannot fully elucidate the damage process of shear deformation throughout the complete cycle of the anchor solid. Consequently, this chapter concentrates on the propagation law of stress waves under the shear deformation of anchor rods and characterizes the shear deformation failure process of anchor solid through this law.

### Noise reduction analysis of stress wave signals based on CEEMD

Due to the existence of various defects, interfaces, grains, and other microstructures within the anchor solid, these microstructures cause nonlinear propagation of the stress wave, and at the same time, due to the fact that the nature of the anchor solid may change with time, temperature, stress, and other factors, resulting in the propagation characteristics of the stress wave changing accordingly, so that the detected signals are non-smooth. Therefore, the use of complete entire empirical modal decomposition (CEEMD) is proposed for nonlinear non-smooth signals to deal with various noises and to help extract local features, dynamic changes, and nonlinear oscillatory components in the signals so as to understand better and analyze the anchor shear stress wave signals.

**Fig. 7:**
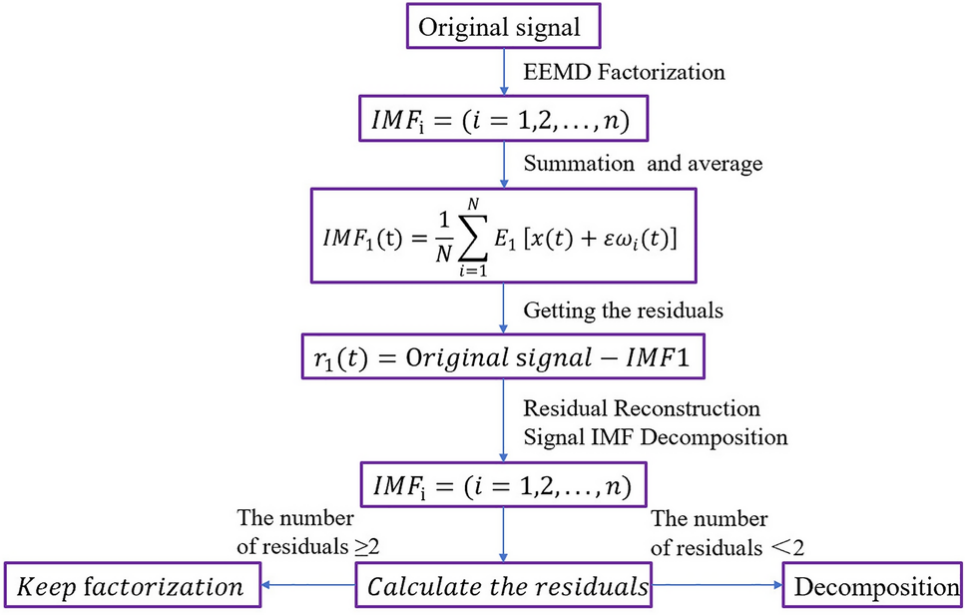
CEEMD decomposition flowchart.

The CEEMD is developed from the empirical modal decomposition (EMD), algorithmic entire empirical modal decomposition (EEMD), EMD decomposition method by the noise-containing signal is decomposed into different modal function IMFs, different IMFs account for a different proportion of the original signal, the proportion of noise-containing IMFs is often tiny, through the reasonable selection of the IMFs to re-compose the signal to achieve the purpose of denoising, but the denoising of the EMD, The disadvantage of EMD denoising, is that it will cause modal aliasing. EEMD is the same as the EMD algorithm, but EEMD adds white noise to the signal on the basis of EMD, which can avoid the modal decomposition, but it will bring the residual noise. CEEMD optimizes the decomposition process on the basis of EEMD, and the CCEMD decomposition not only solves the modal aliasing problem of the EMD decomposition but also avoids the residual noise brought by the EEMD decomposition method. CCEMD decomposition not only solves the modal aliasing problem of the EMD decomposition method but also avoids the residual noise problem brought by the EEMD decomposition method, which is a better means of nonlinear non-smooth signal processing. Figure 7 shows the flow chart of CEEMD decomposition.

The parameters that need to be set for CEEMD modal decomposition are the proportion of added noise and the number of times of summed averaging. When the number of summation averages is fixed, the standard deviation of the error increases with the increase of the proportion of added noise. When the proportion of added noise is certain, the more the number of summation averages, the closer the final result is to the actual signal. According to the experimental debugging results, it is finally determined that the proportion of added noise and the number of times of sum averaging are set to 0.02 and 10, respectively, and it is determined that the decomposition of the IMF modal component is 7, which has the optimal noise reduction effect.

**Fig. 8:**
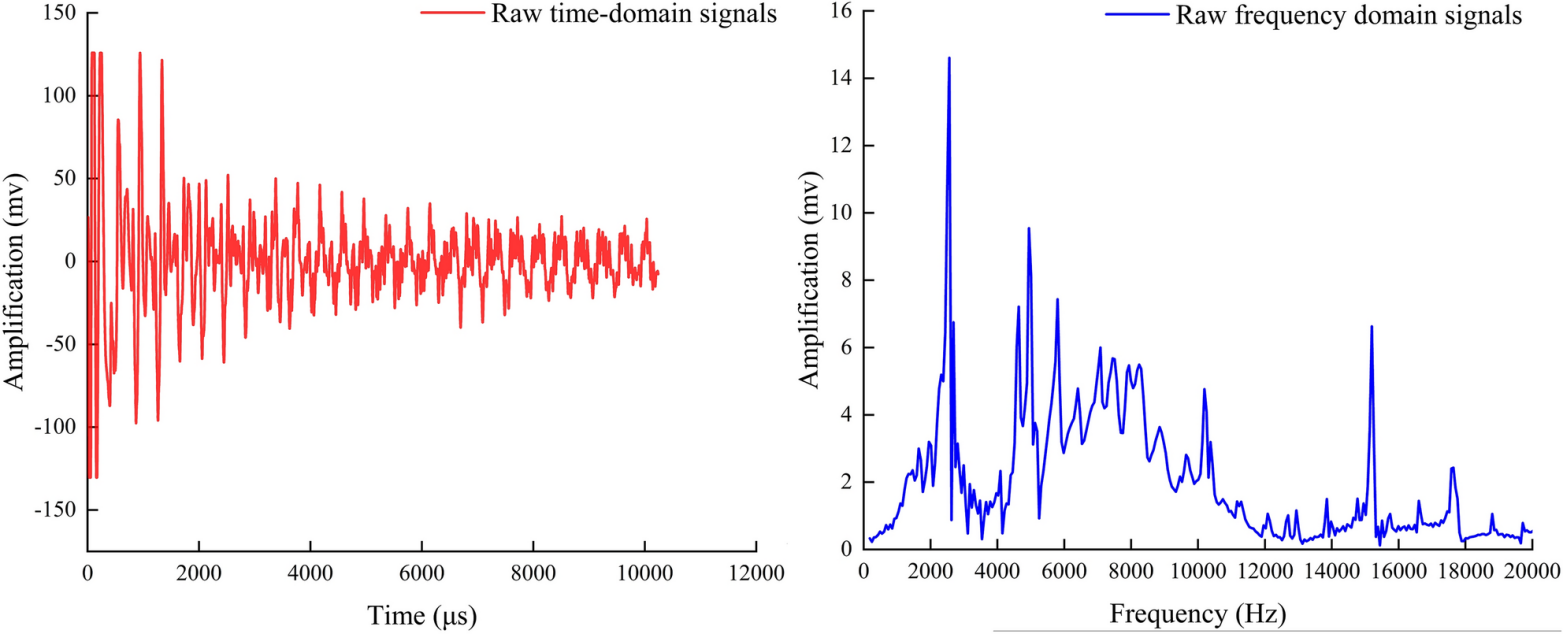
Time-domain and frequency-domain plots of the original signal.

**Fig. 9:**
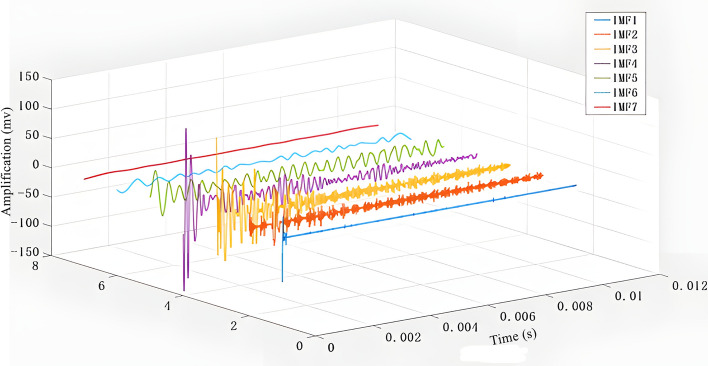
IMF modal component time-domain plot.

**Fig. 10:**
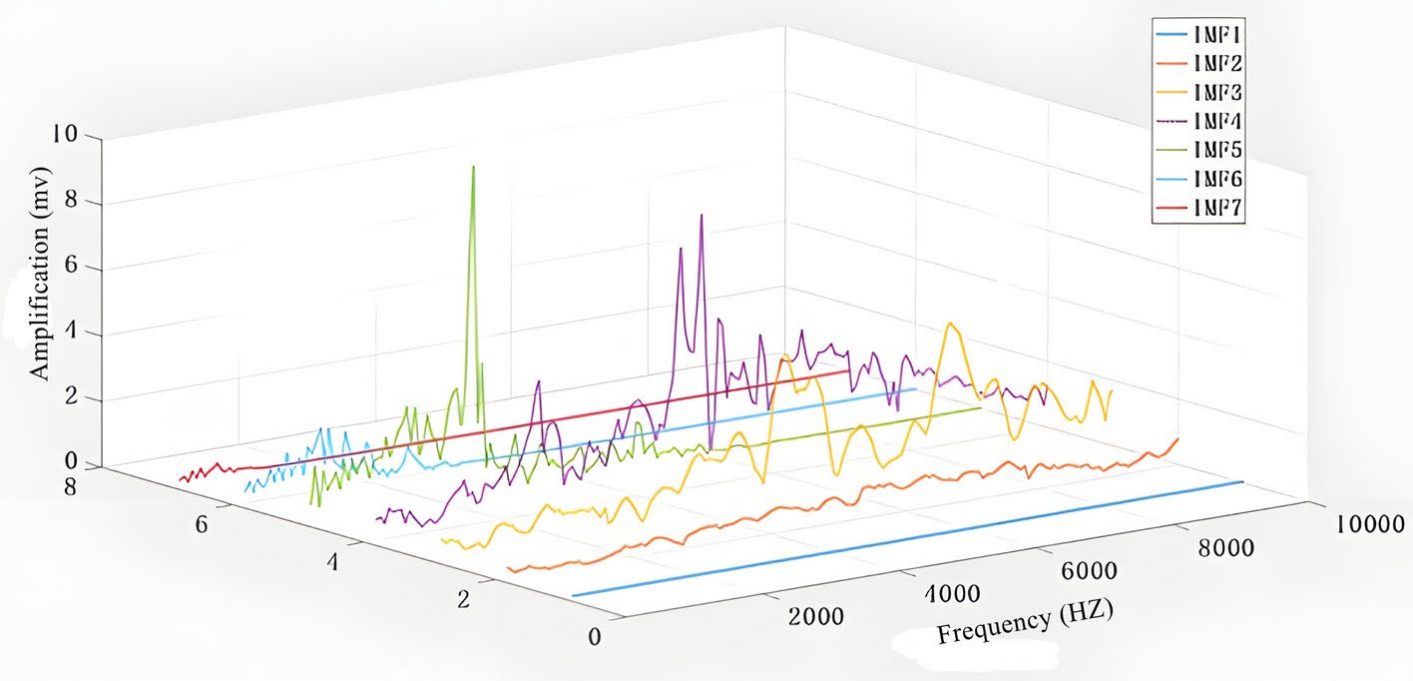
Frequency domain plot of IMF modal components.

Taking the original signal time-domain map in Fig. 8 and the frequency-domain map obtained by Fourier transform of the time-domain map as an example, Figs. 9 and 10 are the time-domain map and frequency-domain map of seven IMF modal components decomposed by the original time-domain map using CEEMD, respectively. Comparative analysis reveals that the amplitude fluctuations of components IMF1 and IMF2 from 1–10000 HZ frequency are minimal, and the difference between high and low peaks is only within 2. The fluctuation of component IMF1 is so small that it is difficult to be seen directly from the plots so IMF1 and IMF2 are the original signal noise. The frequency domain graphs IMF3, 4, 5 and the time domain graph decomposition IMF3, 4, 5 components as well as the original frequency domain graph to maintain a high degree of consistency with the original frequency domain graph, in which IMF3, 4, 5 can represent the original frequency domain graph of the mid-high frequency amplitude, the mid-frequency domain amplitude and the low-frequency domain amplitude, respectively.IMF6 has a small fluctuation in the low-frequency region; the fluctuation is similar to the IMF5, but in the mid-range and high-frequency region, shaped like a straight line, which shows that IMF6 is the decomposition residual.IMF7 frequency fluctuation range is smaller compared to IMF6, so IMF7 is the residual.

**Fig. 11:**
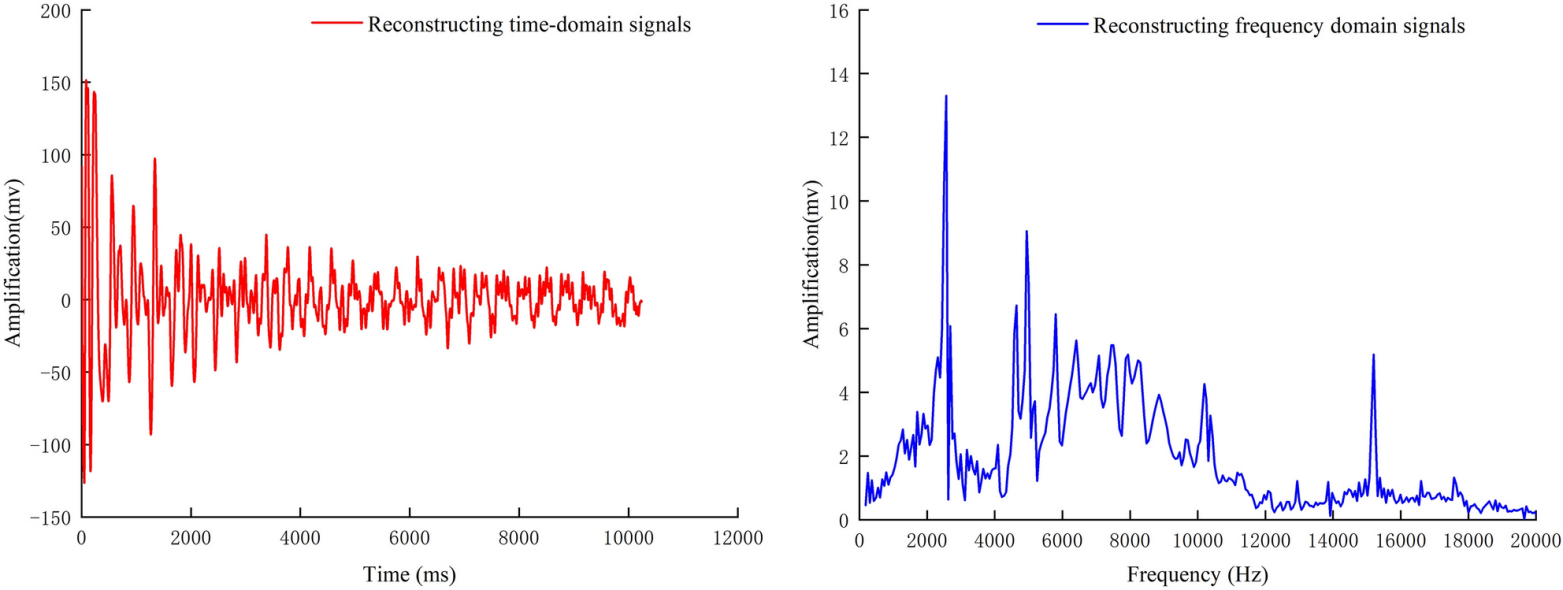
CEEMD reconfiguration signal.

Table 2 shows the results of calculating the number of interrelationships and energy between each IMF component and the original signal, from which it can be clearly seen that the number of interrelationships and energy between IMF3, IMF4, and IMF5 and the original signal are much larger than those of the other components. IMF3, IMF4, and IMF5 basically contain the main features and the primary energy of the original signal. They can be used as the characteristics of the original signal. Components for reconstruction and the reconstructed signal are obtained, as shown in (Fig. 11).

**Table 2:** The cross-correlation coefficient and energy table of each IMF component and the original signal.

Number	IMF1	IMF2	IMF3	IMF4	IMF5	IMF6	IMF7
Common interest	0.010365	0.10852	0.55594	0.70117	0.47448	0.1167	0.00754
Energy	52330	409271	2297396	3221027	1016929	79203	18342

The root mean square error (RMSE) of the original signal and the reconstructed signal are obtained through calculation, as shown in (Table 3). The signal-to-noise ratio of the original signal is lower than that of the reconstructed signal. The RMSE is more significant than that of the reconstructed signal, which indicates that through the complete overall empirical modal decomposition of CEEMD, the reconstructed signals are closer to the predicted value than the actual value of the original signals. The extent of the data deviation is significantly reduced, which removes the noise very well under the precondition of retaining the main features of the signals. In the subsequent signal processing of the article, the CEEMD noise reduction method will be invoked to reduce the noise of the test signal first before signal analysis.

**Table 3:** Noise evaluation index table of original signal and noise reduction signal.

	Original signal	Reconstructed signal
SNR	10.61	18.55
RMSE	14.88	3.39

### Time-domain analysis of internal stress waves in anchors under shear action

**Fig. 12:**
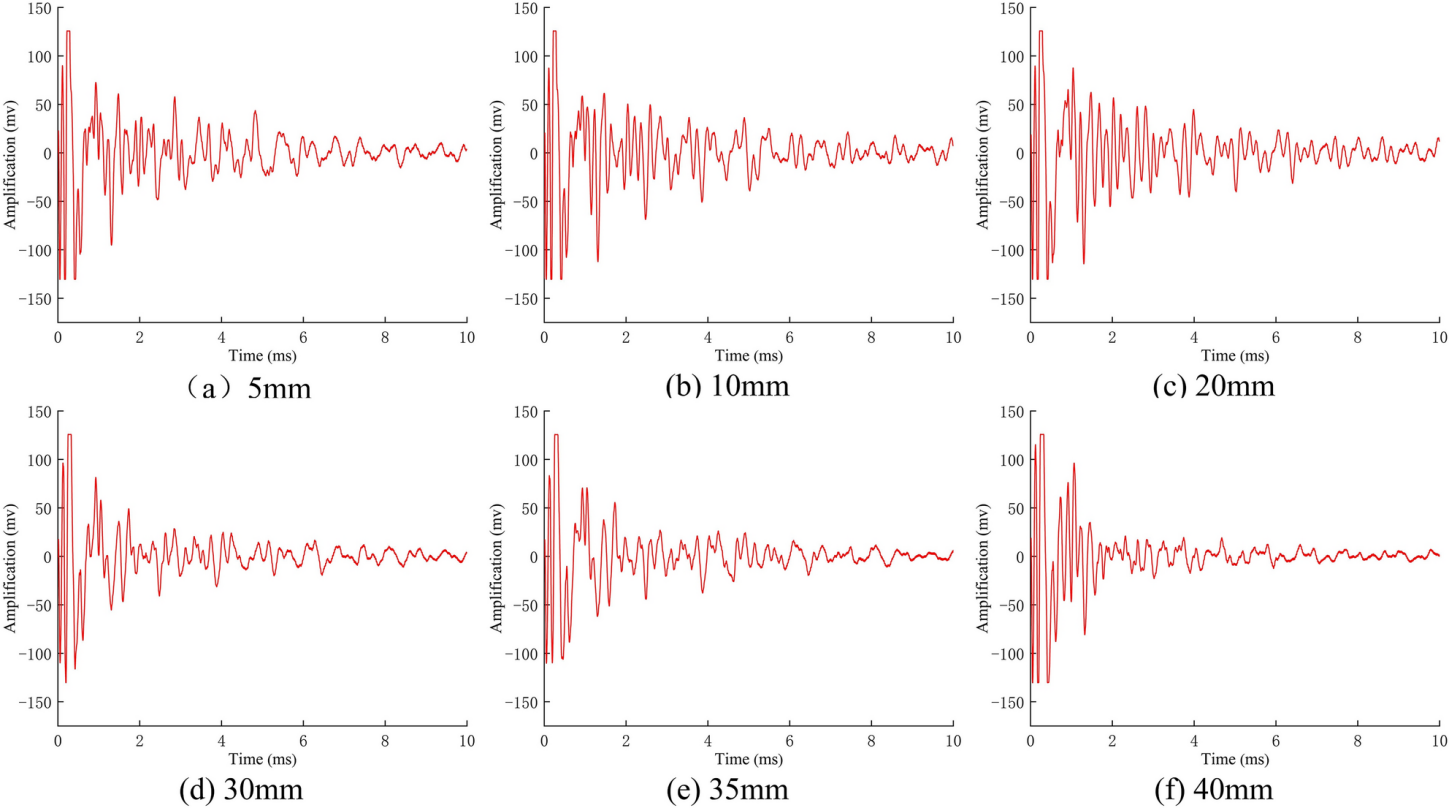
Time-domain signals of stress waves with different shear displacements.

As illustrated in Fig. 12, the original signal from a group of anchor solids, subjected to noise reduction, exhibits a distinct reduction in shear displacement stress wave time domain signal. The horizontal axis of this time-domain waveform is the detection time, and the vertical axis is the waveform amplitude. Comparing the two reveals the change process of the stress waveform’s time-domain waveform amplitude under different shear displacements. Upon examination of the waveforms, it becomes evident that the stress waveforms in the time domain exhibit a high degree of similarity. The peak value of each cycle wave manifests in a relatively consistent manner, and the fluctuation pattern remains largely unaltered. However, in the case of high shear displacement, the amplitude of the stress wave displays a pronounced attenuation phenomenon. Consequently, the statistical analysis parameters of variance and skewness are employed to elucidate the underlying mechanism of the time-domain waveform alterations induced by shear displacement.

The variance (VA) is the average of the squared value of the difference between the mean of each sample value and the mean of all sample values, representing the dynamic component of the signal energy (the square of the mean is the static component), responding to the degree of dispersion between the data, affecting the gap between the high-frequency signals and the low-frequency signals in the time-domain plot; the smaller the gap, the greater the energy attenuation. The calculation formula is:

1 $$VA = \sigma_{x}^{2} (t) = \mathop {\lim }\limits_{N \to \infty } \frac{1}{N}\sum\limits_{i = 1}^{N} {\left[ {x_{i} (t) - \mu_{x} (t)} \right]^{2} }$$

In the formula, N is the signal sample size, which is related to the sampling frequency; $x_{i} (t)$ is the average value of the signal amplitude; and $\mu_{x} (t)$ is the magnitude size of each signal.

Skewness (SK) measures the degree to which the probability distribution of a random variable deviates from the normal distribution, i.e., in a single-peak distribution, the frequency is mainly distributed to the left of the axis as positive and to the right as unfavorable. If the signal peak is shifted to the left, the skewness increases, and the energy is then mainly distributed in the early stage of propagation, calculated as:

2 $$SK = \frac{1}{N}\sum\limits_{i = 1}^{N} {\left[ {(\frac{{x_{i} - \mu }}{\sigma })^{3} } \right]}$$

In the formula, N is the signal sample size, which is related to the sampling frequency; $x_{i}$ is each signal amplitude;$\mu$ is the arithmetic mean of the signal amplitude; and $\sigma$ is the standard deviation of the signal amplitude.

**Fig. 13:**
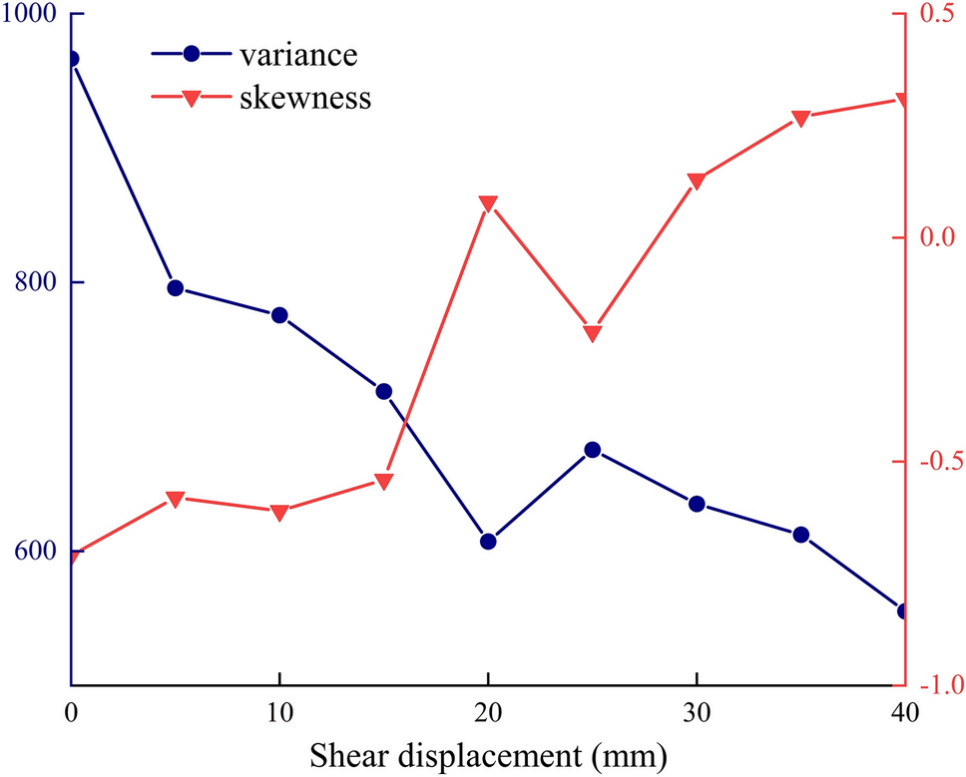
Shear displacement-variance, skewness curves.

As illustrated in Fig. 13, the shear displacement-variance and skewness curves indicate a contrasting trajectory for the variance and skewness as the shear displacement of the anchor solid increases. Specifically, the variance exhibits a decline, while the skewness demonstrates an upward shift. The reduction in variance indicates a decrease in the degree of dispersion of the data, which is reflected in the reduction of the difference between the high and low frequencies of the signal at a given time in the time domain plot. An increase in skewness indicates a decrease in frequency values on the right side of the peak in comparison to the left side of the peak during each peak period in the time-domain signal. The reduction of variance and the increase of skewness can be interpreted as evidence that the energy attenuation of the signal propagated by the stress wave in the anchor is proportional to the amount of shear displacement of the anchor solid. This suggests that as the amount of shear displacement increases, the attenuation of the signal strength also increases.

In light of the conclusions above, the accumulated stress wave time-domain signals are subjected to an energy calculation. Given that alterations to the anchor solid and anchor rod will impact the propagation of the stress wave within the anchor rod following the attainment of a certain degree of shear displacement, the approach of identifying the bending point through the calculation of the stress wave’s velocity is not a viable method. Upon examination of the time-domain diagram of the stress wave, it becomes evident that a significant attenuation of frequency occurs at the 5 ms mark. Consequently, the 5 ms point is designated as the node, and the energy before and after this point is calculated to determine the energy loss ratio. The energy loss ratio formula is as follows (where E1 is the energy of the last 5 ms and E2 is the energy of the first 5 ms):

3 $$E_{\partial } = 1 - \frac{{E_{1} }}{{E_{2} }}$$

The field of anchor NDT is mainly researched from the perspectives of stress wave period, amplitude, multi-scale entropy, and so on. From the above stress wave time domain waveform is not difficult to find; there is an obvious periodicity of the stress wave. Predoi et al.^19^ through the analysis of the periodicity of the stress wave change rule, proposed that the damage to the anchor body will have an impact on the periodicity of the stress wave law, but the periodicity of the material itself by the nature of the impact is more obvious, and can not be applied to non-homogeneous, the material anisotropy is obvious in the anchor solid; Therefore, Li et al.^20^ have proposed to detect the anchor solid according to the stress wave amplitude, which can characterize the change process of the stress wave in the propagation process by defects, deformation, anchoring agent, and other factors, but there is a lack of mature quantitative indexes to characterize the process, and it can only be used in the detection of anchoring defects and anchoring length in the anchoring state of stable anchorage; In addition, Zhang et al.^21^ have proposed using the multi-scale entropy analysis method to portray the degree of change of reflection signals by measuring their complexity and then giving the characteristics of the damage distribution of the anchor solid. This requires the establishment of interrelationships between multi-featured parameters, and there are many difficulties in field practice. Moreover, the energy decay ratio proposed in this paper, from the relationship between stress wave amplitude and energy, to establish quantitative indicators to characterize the change process of stress wave amplitude, to a certain extent, to solve the amplitude can not be applied to anchor solid shear damage deformation detection problems.

**Fig. 14:**
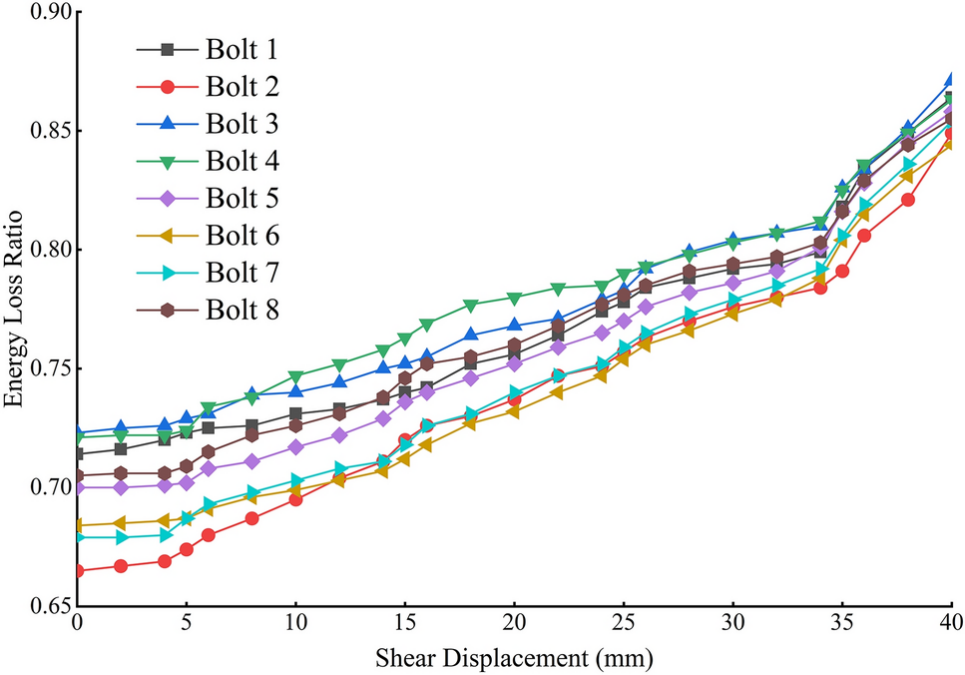
Plot of shear displacement and energy loss ratio of stress waves.

As illustrated in Fig. 14, the shear displacement is found to be directly proportional to the energy loss ratio of the stress wave during the application of shear force to the anchor. The energy loss ratio of the stress wave with shear displacement within 5 mm exhibits a change of approximately 0.02. During this period, the majority of the shear deformation of the anchor solid is borne by the surrounding rock, while the deformation of the anchor rod is minimal. During the 5–35 mm shear displacement process, the anchor solid nodal section and extruded rupture section exhibit significant bending in numerous locations, resulting in a downward bending of the entire anchor rod. Additionally, the gradual rupture of the anchoring agent contributes to the generation of reflected and refracted waves within the anchor rod, leading to an increase in energy loss. When the shear displacement exceeds 35 mm, the anchor enters the stage of plastic reinforcement, accompanied by the emergence of cracks and necking phenomena. The stress wave encounters these cracks and necking when reflection and transmission are more intense, resulting in a faster rate of energy attenuation compared to that observed between 35 and 5 mm.

The conclusion is similar to the research results of many scholars; Niu et al.^22^ measured the amplitude attenuation of the stress wave propagation of the full-length undamaged anchor solid to be about 65%, which is consistent with the energy attenuation law obtained in the experimental unsheared state, and can verify the accuracy of the experimental results,Zhang et al.^21^ judge the anchorage quality and anchorage defects by establishing the energy ratio parameter k, which is the ratio of the reflected energy of the stress wave to the incident energy, and it does not involve the study of anchor deformation, Based on the above research results, this paper characterizes the deformation process of anchor rods, establishing and perfecting the energy loss ratio of stress waves. This broadens the application scope for stress wave anchor NDT technology.

### Numerical simulation analysis of stress wave propagation in anchors under shear action

Due to the complexity and unobservability of the stress wave propagation process in laboratory experiments, it is impossible to accurately describe the propagation process of the stress wave in the shear deformation of the anchors by laboratory experiments, so it is necessary to use numerical simulation methods to observe and verify the accuracy of laboratory experiments. Therefore, based on the numerical simulation model in Chapter 1.3, a small hammer is added to apply excitation to the anchor solid specimen, and other numerical models remain unchanged. In order to elucidate the propagation pattern of the stress wave in the anchor prior to and following shear, the global stress maps of varying moments in the stress wave’s propagation within the anchor are extracted and analyzed.

**Fig. 15:**
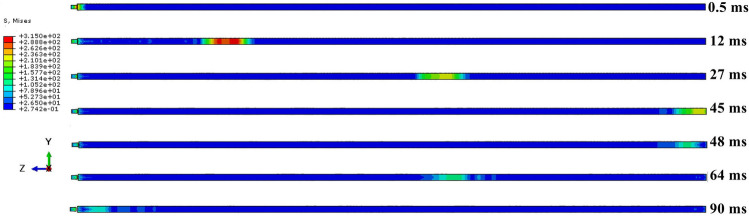
Unsheared stress wave propagation cloud.

**Fig. 16:**
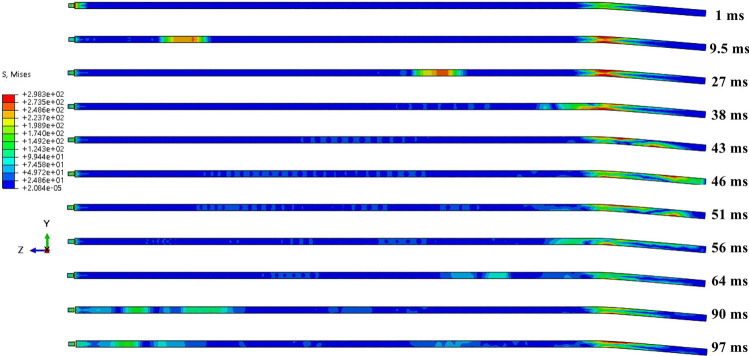
Stress wave propagation cloud with 20 mm shear.

As illustrated in Figs. 15 and 16, the stress wave propagation maps for the unsheared anchor with a shear displacement of 20 mm demonstrate that the propagation is relatively smooth, with minimal energy loss from the bottom of the reflection at 48 ms. The first propagation period is 89 ms. The propagation of the stress wave in the free end of the anchor at a shear displacement of 20 mm is analogous to that observed in the unsheared case, wherein the stress wave transitions into shear. Upon entering the shear area due to the partial bending deformation of the anchor bar, the stress wave is divided into several independent signals (primary wave and secondary wave) during its backward propagation. These signals reach their lowest point and are reflected at 46 and 51 ms, subsequently traversing the shear area once more. A comparison of the stress cloud before and after the shear area reveals that the signal of the stress wave is significantly attenuated, and some of the reflected signals are no longer discernible within the cloud. Following reflection, the primary wave reaches the end in 90 ms, which is in close proximity to the propagation time of the stress wave in the absence of shearing. This suggests that the intrinsic nature of the anchor remains unaltered mainly under conditions of 20 mm shearing and that the velocity of the stress wave within the anchor does not undergo a sudden change. However, the influence of the shear zone results in the secondary wave reaching the end at 97 ms, which is affected by the shear zone on numerous occasions. Its wave speed is considerably less than that of the primary wave, and the energy attenuation is more pronounced.

**Fig. 17:**
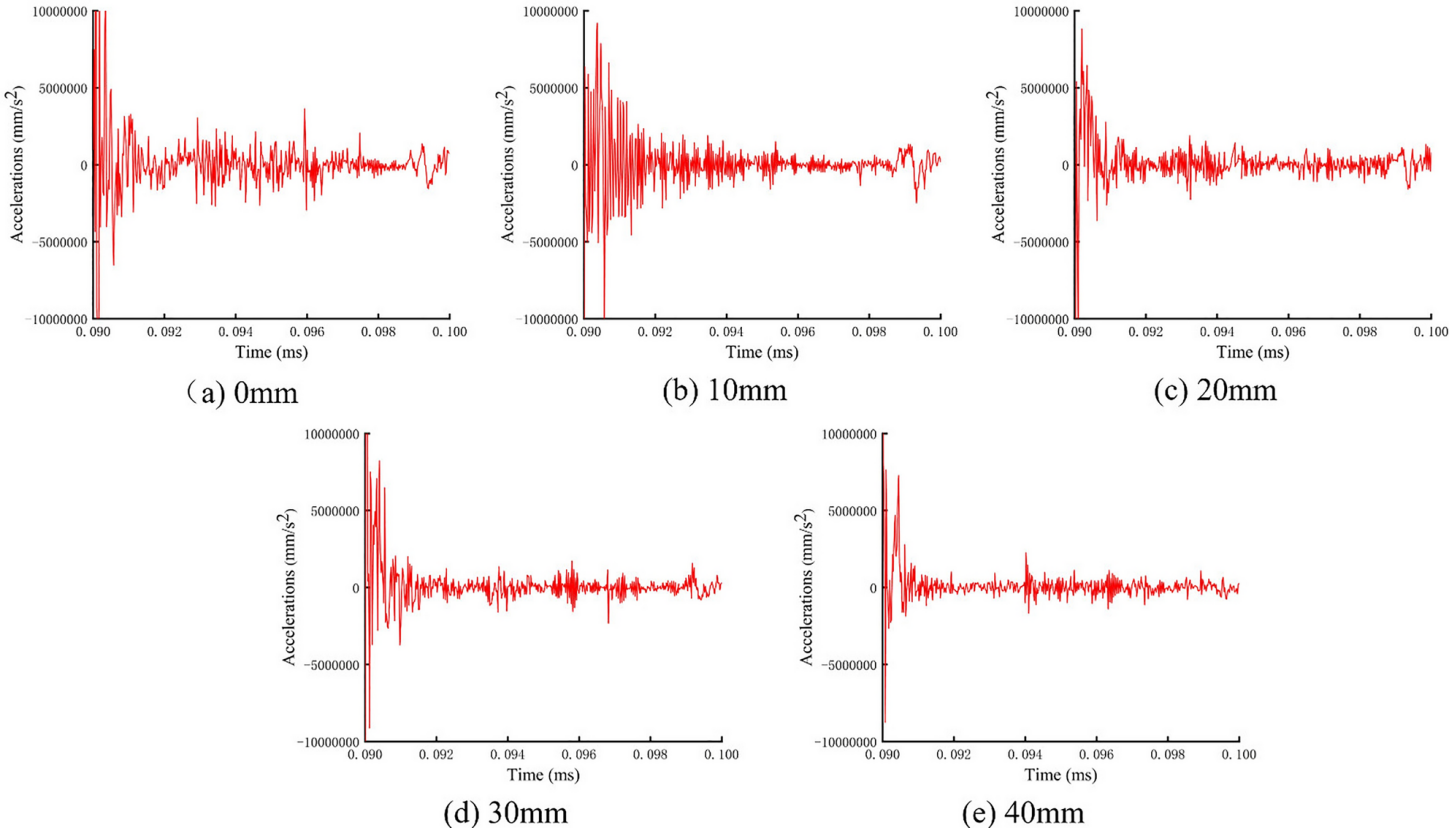
Acceleration-time curves at different shear displacements.

In order to ascertain the veracity of the experimentally obtained stress wave energy loss law under disparate shear displacements of the anchor solid, the acceleration-time curves under varying shear displacements at the left endpoint of the anchor were extracted and is presented in (Fig. 17). A comparison of the laboratory measurements with the aforementioned results reveals that as the shear displacement of the anchor solid increases, the acceleration of the stress wave in the anchor decreases more markedly, and the energy attenuation is accelerated. The trend of energy decay ratio between the two is identical, and the numerical simulation results are consistent with the laboratory tests.

## NDT method and experimental validation of stress wave for anchor shear deformation

### Analysis of factors affecting the energy evolution of variable stress waves in anchors under shear action

As demonstrated by the preceding analysis, the factors influencing the stress wave propagation pattern within the anchor during the anchor solid shear test include the diameter of the anchor, the magnitude of the internal stress of the anchor during the shear process, and the bending angle of the anchor. It is thus imperative to ascertain which factor exerts the dominant influence. In the analysis of the shear deformation characteristics of the anchor rods, only Bolt1 and Bolt8 exhibited a necking phenomenon accompanied by a change in diameter. The change in diameter of the anchor rods is not the cause of the evolution of the stress wave energy.

**Fig. 18:**
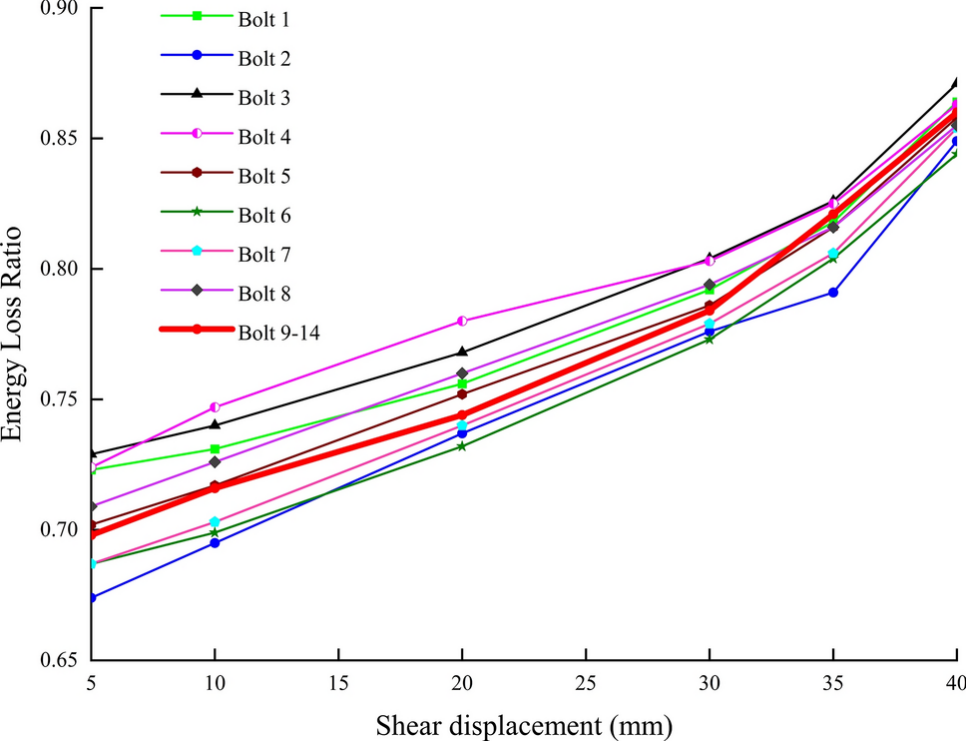
Energy decay law under different stress changes.

To determine whether the presence of stress during the shear process results in a change in stress wave energy attenuation in the anchor rods, a series of experiments involving Bolt9-14 was conducted. The experiments entailed unloading the anchor and subsequently detecting the stress wave. The shear displacements of the rods reached 5, 10, 20, 30, 35, and 40 mm, respectively. Subsequently, the energy evolution laws of Bolt9-14 were compared with those of Bolt1-8. The results of the calculations are presented in (Fig. 18). It can be observed that the energy loss ratio curve of Bolt9-14 with varying anchor solid shear displacement is mainly consistent with that of Bolt1-8. The stress wave data for Bolt1-8 was obtained at the conclusion of the shear test, which was conducted concurrently with the detection of the stress wave data for the anchor solid undergoing shearing. The stress wave data for Bolt9-14 was similarly detected at the conclusion of the shear test. The presence of internal stress within the anchor does not affect the energy evolution of the stress wave within the anchor.

**Fig. 19:**
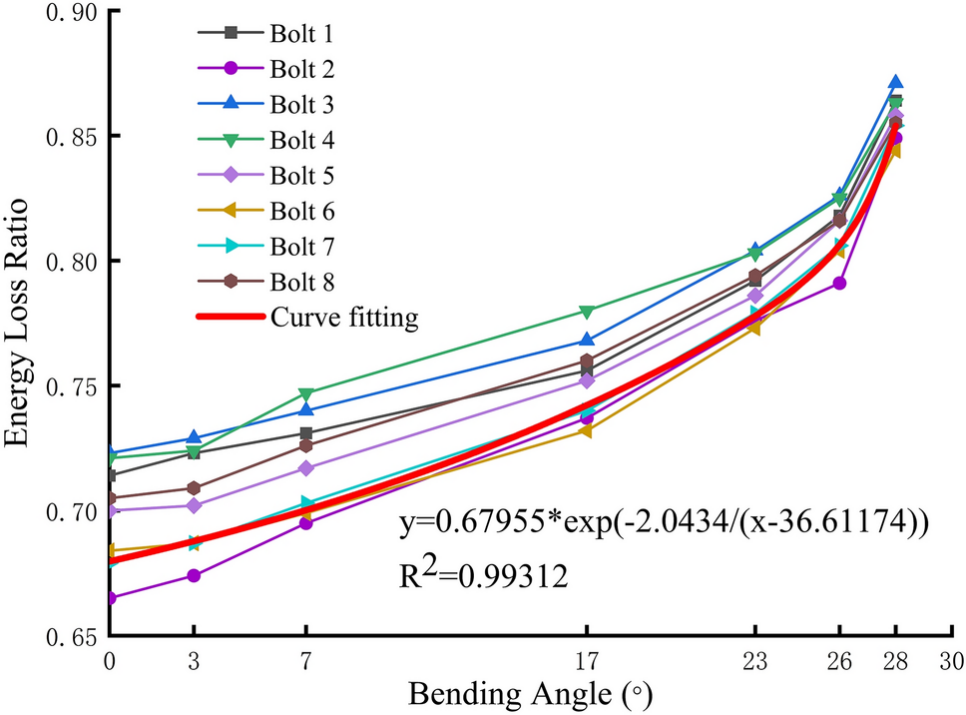
Anchor bending angle-stress wave energy loss ratio relationship and fitting curve.

As illustrated in Fig. 19, the correlation between the bending angle of the anchor bar and the energy loss ratio of the stress wave is depicted. It can be observed that the energy loss ratio of the stress wave exhibits a general tendency to increase with the increase of the bending angle. Prior to the 23° bending angle of the anchor, the energy loss ratio exhibits linear growth. Additionally, the initial energy loss ratio of the anchors differs considerably. Subsequent to the 23° bending angle, the energy loss ratio growth rate accelerates. Concurrently, the difference in the initial energy loss ratio of the anchors diminishes. This phenomenon is attributed to two factors: (1) The anchor solid shear displacement-energy loss ratio curve exhibits a rapid increase in energy loss ratio at a shear displacement of 35 mm. This phenomenon can be attributed to two factors: (1) the anchor solid shear displacement-energy loss ratio curve demonstrates a pronounced rise after reaching a shear displacement of 35 mm; (2) the anchor solid shear displacement-anchor bending angle curve displays a gradual decline in the influence of anchor solid shear displacement on anchor bending angle after a shear displacement of 20 mm.

### Anchor shear deformation detection method based on stress wave propagation characteristics

Figure 19 illustrates the data points obtained through function fitting for the anchor bending angle and stress wave energy loss ratio. Due to the relatively small bending angle, there is a slight deviation in the initial energy loss ratio of the anchor. The function exhibits a slight offset, but as the anchor bending angle increases, it aligns more closely with the data points, effectively capturing the change in the energy loss ratio of the stress wave under shear deformation. The fitting formula can predict the bending angle of the anchor according to the anchor excitation stress wave signal, reflecting the degree of deformation of the anchor, thus realizing NDT of the anchor in the working condition.

Accordingly, the stress wave anchor NDT method, based on the anchor bending angle curve and the deformation characteristics of the anchor under shear, classifies the anchor’s deformation state into three categories by taking the bending angle as a criterion. The proposed evaluation criteria for the NDT of the anchor’s deformation state under shear are presented in (Table 4).

**Table 4:** Evaluation criteria for non-destructive testing of bolt deformation state under shear action.

Bending angle (°)	Operating condition
0–7	Slow growth of anchor bending angle under shear, normal working condition
7–23	Anchor bending angle growth accelerated under shear, normal working condition
> 23	The bending angle of the anchor rod increases rapidly under the shear action, and it is very easy to enter the necking stage. The overall support strength of the anchor decreases under the necking condition and breaks after reaching the limit, at this time the anchor working condition is abnormal

### Experimental validation of anchor deformation detection under shear action

In order to ascertain the veracity and viability of the exponential function derived through fitting, an experimental verification process is conducted in a laboratory setting. By determining the energy decay ratio of the stress wave of the anchor solid at different shear displacements and calculating the bending angle of the anchor at this time based on the fitted curve. Subsequently, the obtained value is compared with the measured anchor bending angle, and the discrepancy and error rate between the two are calculated.

As illustrated in Table 5, the results of the verification experiment and the corresponding error table are presented. A comparison of the measured values of the anchor bending angle and the results of stress wave detection indicates that the maximum discrepancy between the two is 1.2°, with an average difference of 0.64° and an average error of detection of 4.3%. When the bending angle is small, the crushing of surrounding rock makes the anchor force uneven, and the error of stress wave energy attenuation ratio is large; as the bending angle of the anchor increases, the anchor force tends to be stable, and the detection error is gradually narrowed, which indicates that the stress wave detection method is more accurate in detecting large deformation anchors, and therefore the existence of fluctuations in the error rate does not affect the accuracy of the detection method. However, in the field detection process, it is still necessary to carry out at least three inspections on the same anchor rod and to compare and analyze the adjacent anchor rod detection data to attenuate the influence of detection error.

**Table 5:** Verify the experimental test results and errors.

Specimen number	Anchor bending angle measurement results (°)	Stress wave method measurement results (°)	Difference (°)	Detection error (%)
YZ1	29	28.4	0.6	2.1
YZ2	21	22.2	1.2	5.4
YZ3	17	15.9	1.1	6.3
YZ4	15	14.8	0.2	1.3
YZ5	11	10.3	0.7	6.4

## Conclusion

In this paper, for the complex problem that the deformation state of an anchor under shear can not be judged, starting from the laboratory test numerical simulation, we studied the characteristics of anchor deformation under shear. The propagation law of stress wave in the anchor with different degrees of deformation under shear and put forward the NDT method of the degree of anchor deformation under shear based on the relationship between the two, which was successfully verified in the validation test at the end. The main conclusions of this paper are as follows:

1. The damage process of surrounding rock and anchoring agent in the anchor body under shear was found, and the deformation characteristics of anchor rods under shear were analyzed. The anchor’s shear deformation mainly shows the anchor’s bending deformation and the diameter reduction in the late stage of the bending deformation. The anchor’s bending angle is directly proportional to the shear displacement of the anchor solid, and the overall trend is slow-fast-slow.
2. The influence law of the deformation of anchor rods under shear on the propagation characteristics of stress waves in anchor rods is revealed. Using the CEEMD signal processing method to reduce the noise of the stress wave signal, we analyze the influence of the deformation characteristics of the anchor bar on the propagation characteristics of the stress wave in the anchor bar and propose to characterize the degree of energy attenuation of the stress wave with energy loss ratio when the anchor bar is subjected to different degrees of deformation under shear.
3. Based on the influence of the deformation characteristics of anchor rods on the propagation characteristics of stress waves under shear, a NDT method for anchor rods’ shear deformation based on the stress wave method is proposed. Through laboratory verification, the overall error of the detection method is small and stable.

## Deliberations

At this stage, this paper has completed the theoretical research and laboratory verification, theoretically demonstrated the feasibility of the field application of anchor shear nondestructive testing technology, but wants to promote the field application, still needs to complete the following work:

1. Due to the experimental conditions, the shear box and shear displacement settings are different from the shear failure process of anchor rods in the field and cannot completely simulate the slip shear process of the real rock layer. The next step will be to modify the experimental equipment to make it more compatible with the field’s geological conditions.
2. Field validation experiments need to be further carried out. This testing method is currently in the theoretical and laboratory research stage, although a large number of verification experiments have been conducted in the laboratory to meet the accuracy of mine anchor detection and portability requirements due to the complexity of the field mine environment, and has a certain degree of danger, the need for a complete technical program and operational means of experimental protection, and then carry out on-site validation experiments.
3. Field testing should meet the mine rules and regulations under the premise of strict compliance with the testing technology program according to the testing program needs to develop the number of detections, detection density, and detection frequency according to the local conditions to select the detection location and stress wave exciter; in the post-processing of the data, taking into account the impact of the error and geological conditions, reasonable screening and processing of the test data, the summary of the formation of a complete roadway anchor deformation NDT report to guide the production of working face.

## Data Availability

The datasets used and/or analysed during the current study available from the corresponding author on reasonable request.
